# Discovery science, an ally in the fight against antimicrobial resistance

**DOI:** 10.1016/j.ebiom.2022.103975

**Published:** 2022-03-24

For the past 2 years, reporting on the COVID-19 pandemic has overshadowed another major health challenge that is threatening to undo a century of progress in medicine. Antimicrobial resistance (AMR)–the ability of bacteria, viruses, fungi, and parasites to resist drugs that are designed to kill them–would not only prevent us from treating specific infectious diseases, but would also affect our ability to do many medical procedures, such as chemotherapy or organ transplantations. As highlighted earlier this year in the Lancet, this problem is already affecting millions of lives in all regions of the world. In this systematic analysis of the global burden of AMR, the Antimicrobial Resistance Collaborators estimated that a median of 1.27 million deaths in 2019 were directly attributable to AMR; a figure that is comparable with deaths from HIV and malaria combined.

The origins of AMR are multifactorial, with one of the largest causes being misuse and overuse of antimicrobials in humans, in animals (especially for farming), and in environmental scenarios. Inadequate measures for infection prevention, and insufficient adherence to treatments, are also key drivers of this problem. This explains that only a One Health approach (a collaborative, multisectoral, and transdisciplinary approach), as proposed by WHO, the Food & Agriculture Organization, and the World Organization of Animal Health, can succeed in preserving the efficacy of present and future drugs. Although policy makers, industry leaders, funding agencies, and health-care workers have a role in this fight, biomedical researchers can support them by filling knowledge gaps or proposing new therapeutics and diagnostic tools.

Recent studies published in *The Lancet* journals showed how research can help to identify the optimal selection, dose, and duration of the existing antimicrobial treatments to achieve the best clinical outcomes for patients, while avoiding increasing risk of drug resistance. The ARTIC PC trial reported in The Lancet in October showed that amoxicillin, an antibiotic commonly prescribed for children with uncomplicated lower respiratory tract infections is, overall, unlikely to be effective and should not be prescribed unless pneumonia is suspected. Once such evidence-based guidelines are defined, political action, in the form of antimicrobial stewardship interventions, are needed to encourage their adoption. For example, in the Netherlands, although guidelines recommend that adults who are hospitalised with moderately severe community acquired pneumonia in non-intensive care unit wards be treated with narrow-spectrum antibiotics, broad-spectrum antibiotics are still frequently used instead. The CAP-PACT study group reported in the Lancet Infectious Diseases the impact of an antimicrobial stewardship intervention trialled in 12 Dutch hospitals. Through clinician education, prospective audits, feedback on antibiotic use, and engagement of opinion leaders, broad-spectrum antibiotic use was safely reduced in patients who partook in the study. However, this type of intervention might not be sufficient on its own to curtail AMR, as suggested by a study done by Shirin Aliabadi in England on the effects of the NHS Quality Premium stewardship programme. Although this intervention, which was based on financial incentives, successfully helped to reduce broad-spectrum antibiotic usage, it simply attenuated the incidence of *Escherichia coli* bacteraemia resistance over a limited period of time. To understand this unhindered spread of AMR, Shirin Aliabadi and colleagues encouraged the community to further investigate the resistome (the genes driving resistance) by asking about their distribution, how they are introduced into the population, and how they are transmitted. In addition, a study of these genes involved in antibiotic-resistance could be used to design prescription recommendations, as shown by Stefano Leo and Vladimir Lazarevic and their colleagues in eBioMedicine. The authors found that, in patients treated for Gram-negative bacteraemia, shortening the duration of treatment (a common recommendation in antibiotic stewardship programmes) did not result in a decreased abundance of antibiotic-resistance genes.

Besides evaluating the use of existing treatments, biomedical researchers are also developing novel therapeutics. For example, apramycin has been proposed as an improved aminoglycoside antibiotic with superior in vitro activity against a range of highly drug-resistant Gram-negative pathogens, as well as greater efficacy in preclinical pneumonia models. Apramycin is currently undergoing clinical trials as a candidate to treat systemic Gram-negative infections. Moreover, in a preclinical proof-of-concept study published in eBioMedicine Katja Becker and colleagues showed that apramycin could also efficiently treat complicated urinary tract infection and acute pyelonephritis. Because they also observed lower nephrotoxicity compared with the current treatment, gentamicin, apramycin could offer a wider therapeutic window than current treatment.

Even though such studies are promising, we cannot rely on only new antibiotics to stop antibiotic resistance. The discovery of new drugs is technically challenging, and there is still not a sustainable economic model to fund the research and development of these new agents. We hope that The Lancet's call to widen the mission of the Global Fund to Fight AIDS, Tuberculosis and Malaria and to include AMR will be heard and will provide new funding opportunities for researchers. As a direct result of these technical and economic challenges, the thinness of the clinical pipeline has been observed every year since 2017 by WHO and the Pew Charitable Trusts. In 2017, to guide research, WHO also released a list of 12 antibiotic-resistant pathogens that should be considered a priority to tackle. An updated analysis of the clinical pipeline, published in Antimicrobial Agents and Chemotherapy this January confirmed that, despite progress, more action is needed: 76 antibacterial agents are currently in clinical development (28 in phase 1, 32 in phase 2, 12 in phase 3, and four under regulatory evaluation) and 41 of them are targeted WHO priority pathogens. However, only 19 of these antibacterial agents have new pharmacophores, and four of these use new modes of actions compared with marketed drugs. These numbers pale in comparison with the more than 1300 treatments under development to tackle cancer. A reason to stay optimistic is the innovative potential observed in the preclinical pipeline. In their review published in Nature Reviews Microbiology in 2019, Ursula Theuretzbacher and colleagues found that, as of May, 2019, 407 antibacterial projects were underway, with almost half of the projects on direct-acting small molecules, and the remainder being non-traditional approaches (eg, repurposed drugs, engineered phages, microbiome-modifying strategies, vaccines, or antivirulence agents). 135 projects involving direct-acting antibiotics represent novel approaches: antimicrobial peptides, natural products, or LpxC inhibitors that target new ribosomal binding sites, the membrane and cell wall, transcription and translation, gene interference, or metabolism. Another positive point is that most of these preclinical research programmes focus on Gram-negative pathogens, targeting the priority pathogens listed by WHO. However, more work and, crucially, funding are needed for these to be translated into effective therapies.

To fight infections, diagnostic tools are as important as therapeutics. A prompt and accurate diagnosis will help health-care providers to select the most appropriate drug and administer it as early as possible. Diagnostic techniques will also detect resistant germs when they emerge so that an alternative treatment, if available, can be proposed. In addition, the results of these tests constitute important information that can be used to shape public health responses (eg, to define the above-mentioned list of priority pathogens). A potential diagnostic tool to discriminate bacterial from viral infections is the flow cytometric determination of host receptors on the surface of blood leukocytes. The existing tools are effective but are often time consuming, costly, or too complex to be done at the point of care. In the December issue of eBioMedicine, Jari Nuutila and colleagues presented an improved version of their previous flow cytometric methods, with a single-tube setup, which allowed easy and efficient detection of bacterial infections within 30 min. Retrospectively, they estimated that, in their cohort, 38% of antibiotic prescriptions were unnecessary and could have been avoided with the proposed screening test. Another tool was presented in January by Arjun Baghela and colleagues. Using bioinformatics to analyse gene expression profiles of patients in emergency rooms for sepsis, they identified signatures reflecting specific sepsis endotypes that could be used for personalised medicine. These profiles could be observed at admission in the emergency room, before any other diagnosis, representing an opportunity to reduce the use of broad-spectrum antibiotics that are currently administered as a first step to all patients.

In addition to the assessment of strategies to improve antibiotic stewardship and the development of novel therapeutics and of reliable diagnostics at point-of-care as shown above, biomedical research should also be used to fill the innovation gaps outlined by the US Centers for Disease Control and Prevention in their Antibiotic resistance threats report: to improve knowledge of the microbiome (especially how it can be leveraged to prevent or treat infection), improve understanding of AMR in the environment, develop strategies to prevent the spread of resistance, and to develop analytical tools to measure the efficiency of these strategies. *eBioMedicine*, as part of *The Lancet Discovery Science*, will continue offering a platform to publish and discuss essential, early evidence on these topics to guide the fight against the spread of AMR.

